# Insights in the regulation of trimetylamine N-oxide production using a comparative biomimetic approach suggest a metabolic switch in hibernating bears

**DOI:** 10.1038/s41598-020-76346-1

**Published:** 2020-11-23

**Authors:** Thomas Ebert, Johanna Painer, Peter Bergman, Abdul Rashid Qureshi, Sylvain Giroud, Gabrielle Stalder, Karolina Kublickiene, Frank Göritz, Sebastian Vetter, Claudia Bieber, Ole Fröbert, Jon M. Arnemo, Andreas Zedrosser, Irene Redtenbacher, Paul G. Shiels, Richard J. Johnson, Peter Stenvinkel

**Affiliations:** 1 Division of Renal Medicine, Department of Clinical Science, Intervention and Technology, Karolinska Institutet, Stockholm, Sweden; 2 Department of Interdisciplinary Life Sciences, Research Institute of Wildlife Ecology, Veterinary University Vienna, Savoyenstreet 1, 1160 Vienna, Austria; 3 Division of Clinical Microbiology, Department of Laboratory Medicine, Karolinska Institutet, Stockholm, Sweden; 4 Leibniz Institute for Zoo and Wildlife Ecology, Berlin, Germany; 5 Department of Cardiology, Faculty of Health, Örebro University, Örebro, Sweden; 6 Department of Forestry and Wildlife Management, Inland Norway University of Applied Sciences, Campus Evenstad, Koppang, Norway; 7 Department of Wildlife, Fish and Environmental Studies, Swedish University of Agricultural Sciences, Umeå, Sweden; 8 Department of Natural Sciences and Environmental Health, University of South-Eastern Norway, Bø i Telemark, Norway; 9 Institute for Wildlife Biology and Game Management, University for Natural Resources and Life Sciences, Vienna, Austria; 10 Four Paws International, Vienna, Austria; 11 Wolfson Wohl Cancer Research Centre, Institute of Cancer Sciences, University of Glasgow, Glasgow, UK; 12 Division of Renal Diseases, University of Colorado Anschutz Medical Campus, Aurora, CO USA; 13 Department of Renal Medicine M99, Karolinska University Hospital, 141 86, Stockholm, Sweden

**Keywords:** Zoology, Nephrology

## Abstract

Experimental studies suggest involvement of trimethylamine N-oxide (TMAO) in the aetiology of cardiometabolic diseases and chronic kidney disease (CKD), in part via metabolism of ingested food. Using a comparative biomimetic approach, we have investigated circulating levels of the gut metabolites betaine, choline, and TMAO in human CKD, across animal species as well as during hibernation in two animal species. Betaine, choline, and TMAO levels were associated with renal function in humans and differed significantly across animal species. Free-ranging brown bears showed a distinct regulation pattern with an increase in betaine (422%) and choline (18%) levels during hibernation, but exhibited undetectable levels of TMAO. Free-ranging brown bears had higher betaine, lower choline, and undetectable TMAO levels compared to captive brown bears. Endogenously produced betaine may protect bears and garden dormice during the vulnerable hibernating period. Carnivorous eating habits are linked to TMAO levels in the animal kingdom. Captivity may alter the microbiota and cause a subsequent increase of TMAO production. Since free-ranging bears seems to turn on a metabolic switch that shunts choline to generate betaine instead of TMAO, characterisation and understanding of such an adaptive switch could hold clues for novel treatment options in burden of lifestyle diseases, such as CKD.

## Introduction

With a global prevalence of 10–12%, chronic kidney disease (CKD) is a major health burden associated with increased morbidity, mortality, and reduced quality of life^1,2^. When patients with CKD have progressed to end-stage kidney disease they exhibit high cardiovascular (CV) mortality^3,4^. CKD manifests with a progeric phenotype^5^ and the survival of incident dialysis patients is lower than that of patients with most solid-organ cancers^6,7^. Some well-established mechanisms linking CKD with CV morbidity and mortality are inflammation^8^, oxidative stress^9^, cellular senescence^10^ and metabolic dysfunction^11^.

**Figure 1:**
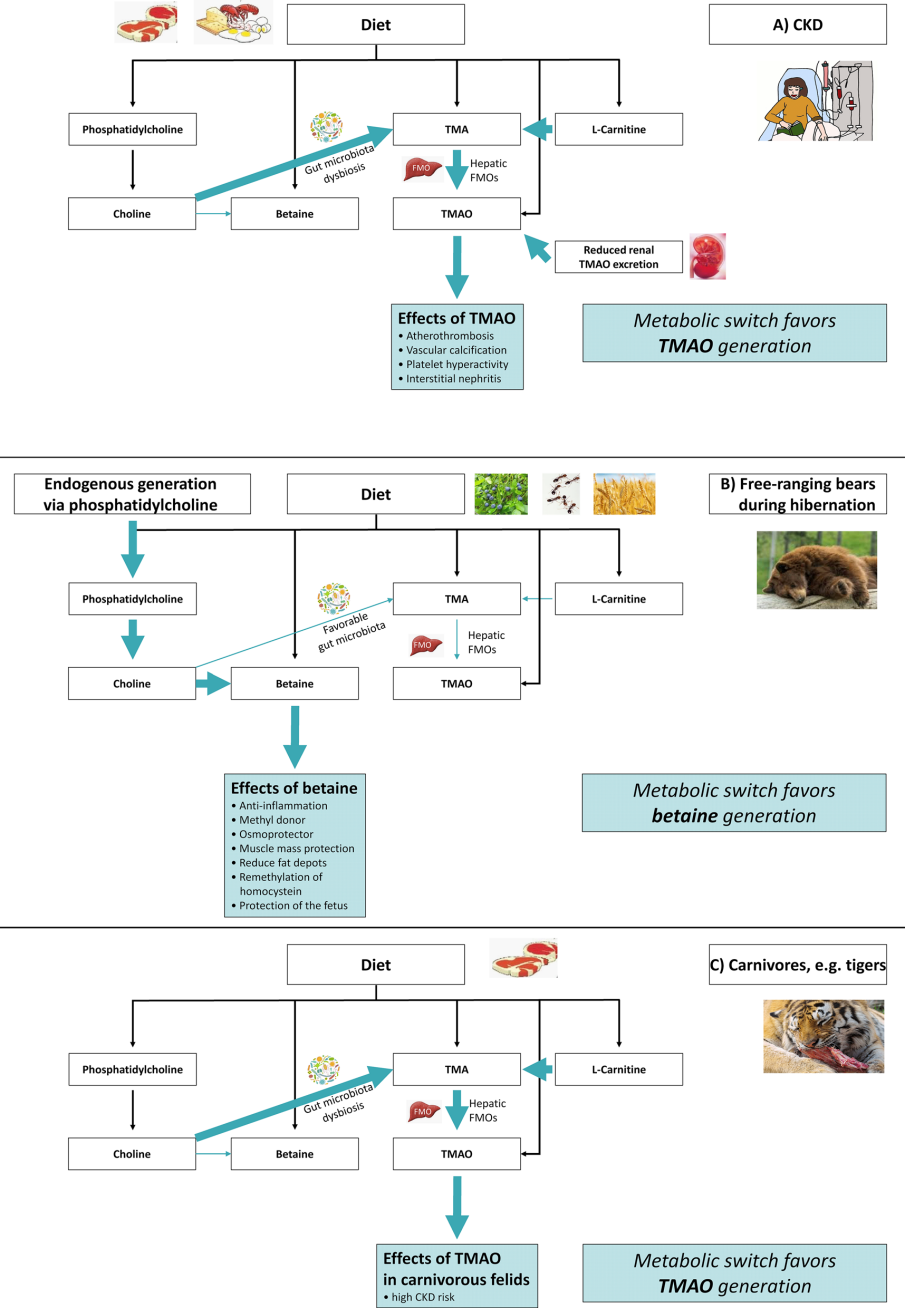
Proposed regulation pattern of betaine, choline, and TMAO in (**A**) patients with chronic kidney disease (CKD); (**B**) CKD-protected, hibernating, free-ranging brown bears; and (**C**) CKD-prone carnivorous felids, e.g. tigers. In patients with CKD (**A**), high levels of trimethylamine (TMA) N-oxide (TMAO) and choline are detectable, whereas betaine levels are low. Dietary intake of phosphatidyl-choline, betaine, TMA, TMAO, and L-Carnitine contribute to circulating metabolite levels. Gut microbiota can metabolize choline and L-Carnitine to TMA. After intestinal uptake of TMA, hepatic flavin-containing monooxygenases (FMOs) convert TMA into TMAO which exerts adverse cardiometabolic effects. TMAO levels in CKD are especially elevated due to an increased microbial TMA conversion (i.e. gut microbiota dysbiosis), as well as a reduced renal excretion of TMAO. In hibernating, free-ranging brown bears (**B**), there is no food intake and betaine, choline, and TMAO are endogenously produced. Based on our data, we propose a metabolic switch favouring betaine rather than TMAO synthesis due to increased betaine conversion from choline and reduced gut microbiota-caused TMA production. In carnivorous animals, e.g. tigers (**C**), dietary intake of phosphatidylcholine, betaine, TMA, TMAO, and L-Carnitine contribute to circulating metabolite levels. Similar to patients with CKD, a metabolic switch favours TMA production by gut microbiota resulting in high TMAO levels that might be involved in the high CKD prevalence of carnivorous felids. Arrow size depicts increased or decreased activation of the respective pathway.

Gut microbes have been associated with CV disease (CVD) and CKD^12^. Although the gut microbiota directly exert critical pathophysiological effects through metabolism of ingested food, it is also self-regulated by dietary habits and distinct disease states^12^. Currently, the trimethylamine (TMA) N-oxide (TMAO) pathway (Fig. 1) is one of the most promising targets for a microbiota-directed treatment approach in several burden-of-lifestyle diseases. Gut microbiota metabolize dietary substrates to produce TMA. The essential nutrient choline can be directly metabolized to TMA, but also to betaine, thereby shunting away from TMAO production^13^. However, if choline production is high, it may result in both increased TMAO and betaine production. Hence, increased betaine could reflect more choline generation and more TMAO production, but could also reflect a relative shunting of choline metabolism to the betaine pathway and away from TMAO production.

Gaseous TMA is absorbed into the circulation and subsequently oxidized to TMAO in the liver by flavin monooxygenases^14^. While high choline and TMAO independently predicted the risk for CVD in patients undergoing elective cardiac evaluations, betaine insufficiency is associated with an adverse vascular risk profile in the metabolic syndrome^15,16^. Moreover, low betaine was associated with components of the metabolic syndrome in healthy men and women^17^ and betaine supplementation reduced body fat^18^. Importantly, TMAO levels are increased in CKD, are associated with mortality risk, and induces renal fibrosis^19,20^. While positive associations with CKD and CVD have been validated especially for TMAO^21–23^, betaine’s and choline’s associations with CVD are mediated by TMAO^24^, a finding recently validated in a meta-analysis^25^.

Based on ingenious solutions developed in some animal species during evolution, we have suggested^26^ that expanding biomedical investigation to new approaches based on a broad awareness of the diversity of animal life and comparative physiology can accelerate innovations in human health care. Natural comparative animal models, such as different nutritional, seasonal and long-time fasting (such as hibernation) patterns and other physiologic adaptions to unfavourable environmental conditions can be used for studying metabolic regulation of the microbiota^27,28^. Here, we investigated circulating levels of the three metabolites betaine, choline and TMAO (1) in patients with CKD stage 3 and 5 and healthy controls; (2) in different animal species with various dietary habits; (3) during hibernation and active periods in free-ranging brown bears and captive garden dormice (*Eliomys quercinus*); (4) in free-ranging brown bears (*Ursus arctos*) and captive brown bears; and (5) cross-sectionally between CKD-prone felids compared to hibernating brown bears and human CKD patients.

## Results

### Betaine, choline, and TMAO in healthy subjects and patients with CKD

**Figure 2:**
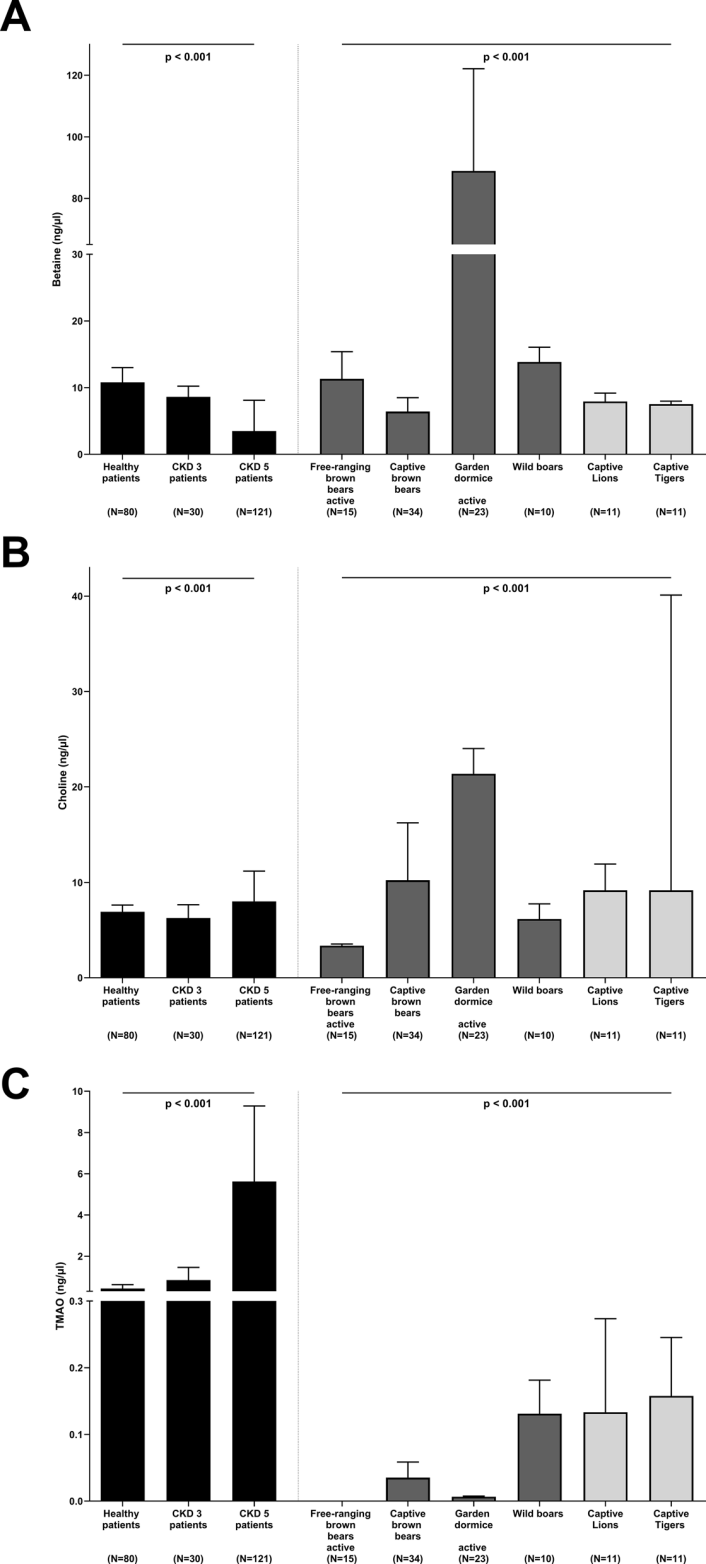
Circulating levels of (**A**) betaine, (**B**) choline, and (**C**) trimethylamine N-oxide (TMAO) in human subjects (black bars) with normal renal function compared to patients with chronic kidney disease (CKD) stage 3 and 5, as well as different animal species, i.e. free-ranging, active brown bears; captive brown bears; captive, active garden dormice; wild boars; captive lions; and captive tigers. Animals are color-coded depending on their diet, i.e. omnivores (dark grey bars), and carnivores (light grey bars). Results are shown as median (interquartile range). Overall *p* values between the three groups of human subjects, as well as between animal species, were assessed by non-parametric Kruskal–Wallis test. Group-wise comparisons using Bonferroni post hoc analysis are described in the Results section. Number of participants or animals included in each group is depicted.

Basic clinical characteristics for healthy subjects and CKD patients have been described previously^20^. In all patients, median [25–75% interquartile range] serum betaine levels were 8.3 [7.8] ng/µl and significantly decreased with deteriorating renal function (*p* < 0.001, Fig. 2A). Thus, patients with CKD 5 had the lowest circulating betaine levels compared to healthy control subjects (*p* < 0.001) and patients with CKD stage 3 (*p* = 0.003, Fig. 2A). Median circulating choline levels were 7.2 [2.7] ng/µl in the entire human cohort. In contrast to betaine, patients with CKD 5 showed the highest choline levels compared to healthy subjects (*p* = 0.07, Fig. 2B) and CKD 3 (*p* = 0.002, Fig. 2B). Serum levels of TMAO in the entire human cohort were 2.3 [5.2] ng/µl. Similar to choline, TMAO significantly increased in kidney disease and patients with CKD 5 had almost 13-fold and sevenfold increased TMAO levels compared to healthy subjects (*p* < 0.001, Fig. 2C) and patients with CKD 3 (*p* < 0.001, Fig. 2C), respectively.

### Betaine, choline, and TMAO levels across species

Betaine, choline, and TMAO levels in active, free-ranging brown bears, captive brown bears, captive garden dormice, as well as wild boars, captive lions, and captive tigers, significantly differed across all species (overall *p* < 0.001). Thus, active dormice showed the highest circulating betaine levels after correction for multiple testing (Fig. 2A). Whereas the lowest circulating choline levels were observed in active, free-ranging brown bears the highest choline levels were found in active garden dormice (Fig. 2B). Notably, TMAO was detectable in only one out of 15 free-ranging bears, but it was measurable in all captive bears (Fig. 2C).

### Hibernation versus active summer state

**Figure 3:**
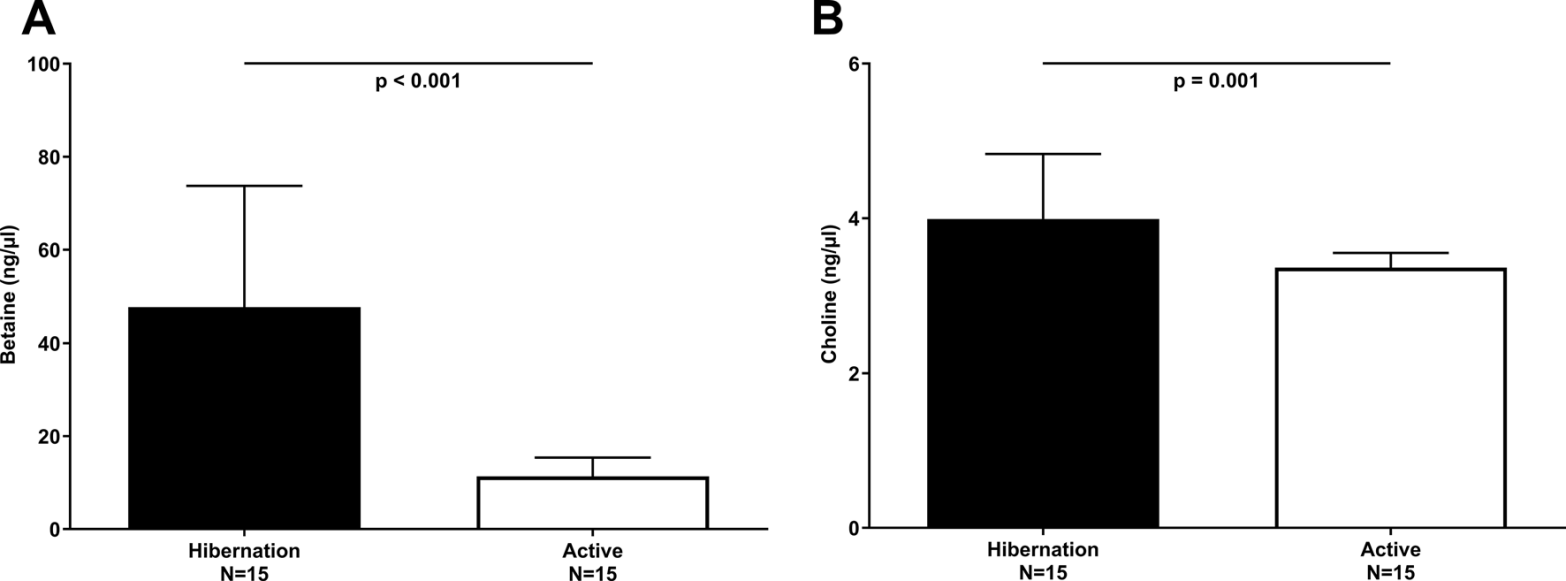
Circulating levels of (**A**) betaine and (**B**) choline in free-ranging brown bears from the longitudinal Scandinavian Brown Bear Research Project during hibernation, i.e. at hibernation (black bars, during winter) and non-hibernation (white bars, during summer). Results are shown as median (interquartile range). *p* values as assessed by non-parametric Wilcoxon signed-rank test. Number of brown bears included in each group is depicted. Aiming to investigate differences between hibernating and active time points, four bears were investigated twice in two consecutive years and treated as independent samples for this figure.

**Figure 4:**
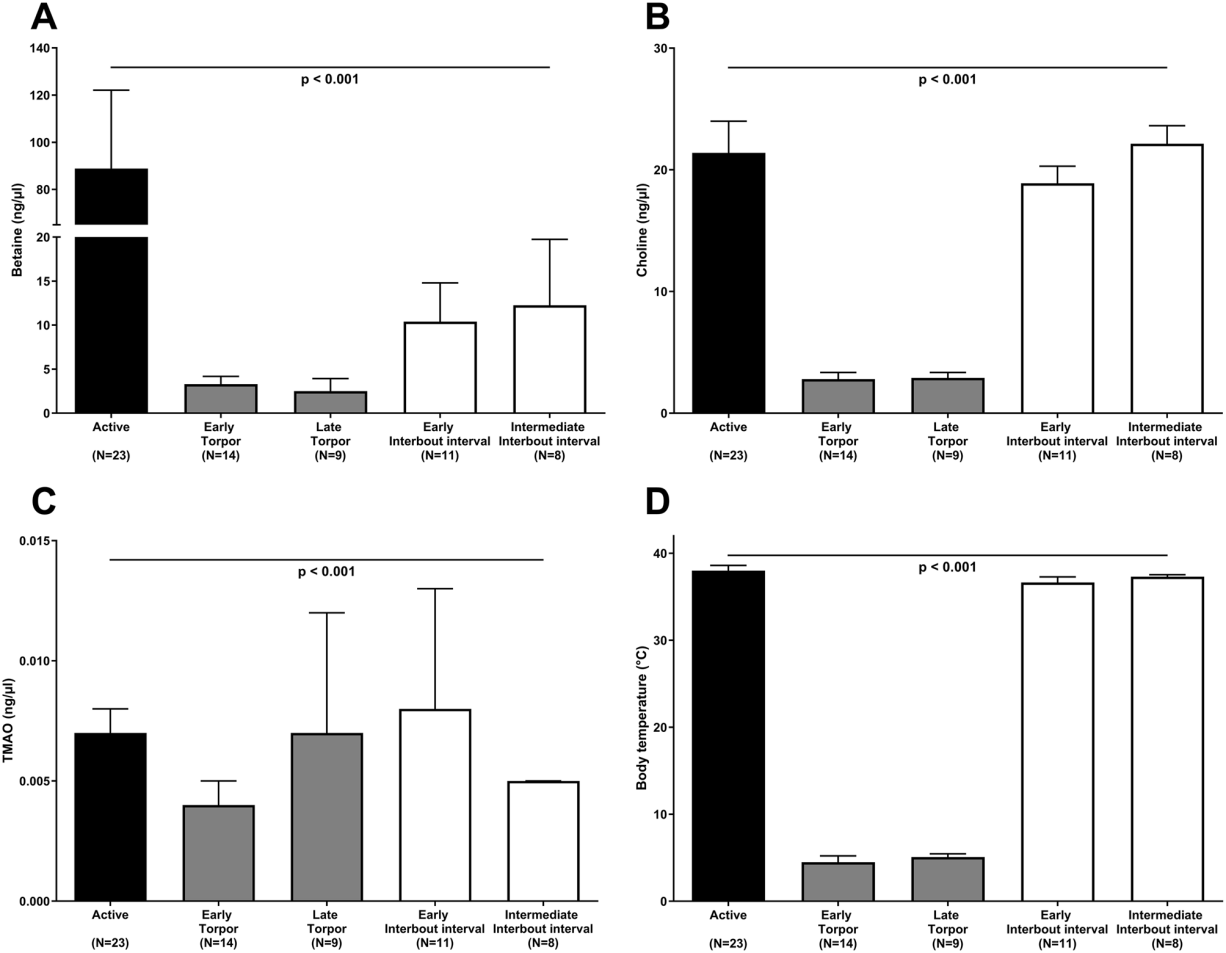
Circulating levels of (**A**) betaine, (**B**) choline, (**C**) trimethylamine N-oxide (TMAO), and (**D**) body temperature in garden dormice at different time points during hibernation, i.e. non-hibernation (black bars), torpor status (grey bars, stratified into early and late torpor), and interbout interval (white bars, stratified into early and intermediate interbout interval). Results are shown as median (interquartile range). Overall *p* values between the five groups were assessed by non-parametric Kruskal–Wallis test. Group-wise comparisons using Bonferroni post hoc analysis are described in the Results section. Number of animals included in each group is depicted.

To investigate the effect of hibernation on betaine, choline, and TMAO levels, the metabolite levels were quantified in two species during hibernation and active periods (i.e. free-ranging brown bears and garden dormice). During hibernation, free-ranging brown bears had significantly (*p* < 0.05) lower body weight, liver enzymes, urea and higher albumin, total protein, triglycerides, creatinine, and insulin levels compared to the active summer period (Table 1). During hibernation, brown bears had 422% higher betaine levels (47.7 vs. 11.3 ng/µl; *p* < 0.001) versus the active summer period (Table 1, Fig. 3A). In contrast, choline levels were only 18% higher in winter (4.0 vs. 3.4 ng/µl; *p* = 0.009) vs. the active summer period (Table 1, Fig. 3B). TMAO levels were below the detection limit in all free-ranging bears during hibernation (Table 1). To investigate if similar changes in betaine, choline and TMAO levels were observed in another hibernating species, we investigated garden dormice. In contrast to hibernating bears, hibernating (i.e. during torpor and interbout interval) garden dormice had significantly lower betaine (4.7 vs. 88.9 ng/µl; *p* < 0.05) (Fig. 4A) and choline (3.6 vs. 21.4 ng/µl; *p* < 0.001) (Fig. 4B) concentrations compared to summer active dormice with a pattern similar to body temperature (Fig. 4D). TMAO levels were low during both hibernation and the active state. Active garden dormice, as well as garden dormice during late torpor and early interbout interval, had higher TMAO concentrations compared to hibernating animals in early torpor and during the intermediate interbout interval (all *p* < 0.05) (Fig. 4C).

**Table 1:** Characteristics of free-ranging brown bears during hibernation and the summer active state.

	Hibernation winter	Active summer	*p*
N	15	15	–
Sex (male/female)	4/11		–
Age at winter sampling (years)	3.2 (1.1)		–
Body weight (kg)	48 (24)	49 (26)	**0.008**
Albumin (g/l)	36.6 (3.4)	30.5 (3.6)	**< 0.001**
Total protein (g/l)	74.0 (5.2)	57.5 (4.6)	**< 0.001**
Calcium (mmol/l)	2.4 (0.1)	2.3 (0.3)	0.268
Phosphate (mmol/l)	1.5 (0.5)	2.4 (0.9)	**< 0.001**
Total cholesterol (mmol/l)	10.4 (5.8)	6.6 (1.3)	0.063
Triglycerides (mmol/l)	4.1 (1.4)	2.1 (1.2)	**< 0.001**
ALAT (U/l)	12.0 (3.8)	35.5 (18.0)	**0.002**
ASAT (U/l)	52.5 (19.8)	89.5 (51.5)	**0.002**
Creatinine (µmol/l)	206 (50)	84 (14)	**< 0.001**
Urea (mmol/l)	3.5 (5.0)	13.6 (6.7)	**< 0.001**
Uric acid (µmol/l)	65 (16)	128 (67)	**< 0.001**
Glucose (mmol/l)	6.9 (2.1)	5.3 (3.4)	0.064
Insulin (mU/l)	2.2 (0.9)	1.5 (1.2)	**0.014**
Cystein (µmol/l)	134.0 (69.3)	149.4 (73.1)	0.330
CysGly (µmol/l)	20.1 (7.4)	24.6 (7.6)	**0.041**
Homocystein (µmol/l)	16.0 (6.8)	11.6 (12.6)	0.229
Betaine (ng/µl)	47.7 (36.5)	11.3 (6.2)	**< 0.001**
Choline (ng/µl)	4.0 (1.4)	3.4 (0.6)	**0.009**
TMAO (ng/µl)	0.0 (0.0)	0.0 (0.0)	–

Characteristics of the free-ranging brown bears during hibernation and the active state, divided into winter (i.e. hibernation) and summer (i.e. active) blood samplings. ALAT, Alanine transaminase; ASAT, Aspartate transaminase; TMAO, Trimethylamine N-oxide. Values for median (interquartile range) or total number are shown. *p* values as assessed by non-parametric Wilcoxon signed-rank test.

### Free-ranging versus captive brown bears

**Figure 5:**
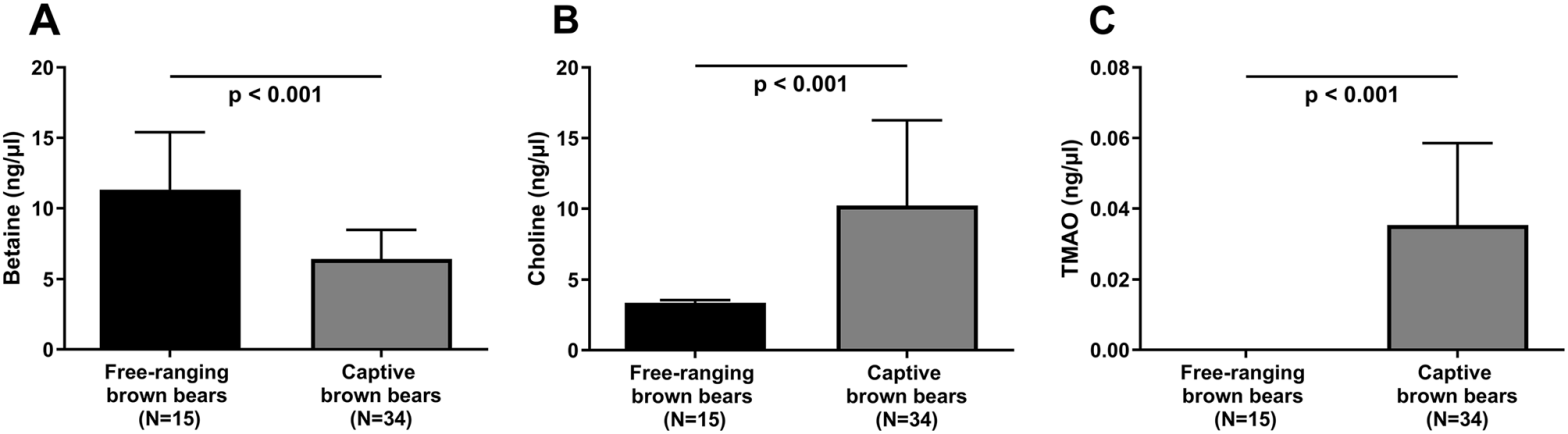
Circulating levels of (**A**) betaine, (**B**) choline, and (**C**) trimethylamine N-oxide (TMAO) in active, free-ranging brown bears (black bars) compared to captive brown bears from European zoos (grey bars). Results are shown as median (interquartile range). *p* values between the two groups were assessed by non-parametric Mann–Whitney U test. Number of animals included in each group is depicted. TMAO levels could only be detected in one out of 15 free-ranging brown bears.

To investigate the impact of captivity, we compared active, free-ranging bears to captive bears. Free-ranging bears had significantly higher betaine levels (11.3 vs. 6.4 ng/µl; *p* < 0.001) and reduced choline levels (3.4 vs. 10.2 ng/µl; *p* < 0.001) compared to captive brown bears (Fig. 5A,B). TMAO could only be detected in one free-ranging bear, but was detected in all captive bears (0.04 ng/µl; *p* < 0.001) (Fig. 5C).

### Comparison between human CKD stage 3 patients and animals with “CKD-like” status

**Figure 6:**
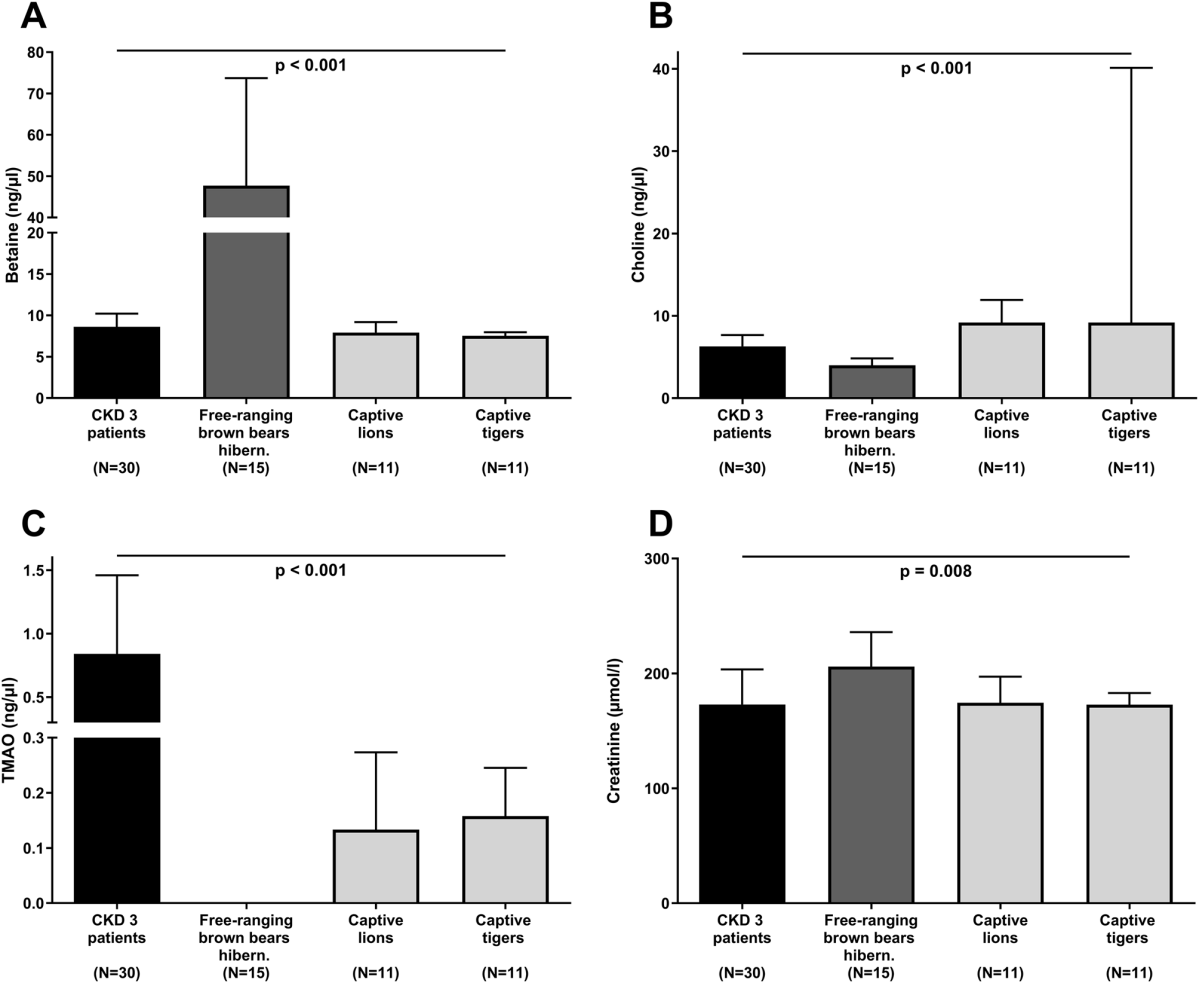
Circulating levels of (**A**) betaine, (**B**) choline, (**C**) trimethylamine N-oxide (TMAO), and (**D**) creatinine in human subjects with chronic kidney disease (CKD) stage 3 (black bars) compared to different animal species with a CKD-like status, i.e. free-ranging brown bears during hibernation (dark grey bars), as well as captive lions and tigers (light grey bars). Results are shown as median (interquartile range). Overall *p* values between the four groups were assessed by non-parametric Kruskal–Wallis test. Group-wise comparisons using Bonferroni post hoc analysis are described in the Results section. Number of subjects/animals included in each group is depicted. TMAO levels could not be detected in hibernating free-ranging brown bears.

Grouped by creatinine levels we compared betaine, choline, and TMAO levels between CKD 3 patients, free-ranging brown bears during hibernation, and felids (Table 2). Whereas felids had betaine levels comparable to CKD 3 patients (Fig. 6A), hibernating brown bears had significantly (*p* < 0.001), fourfold higher betaine levels than CKD 3 patients and felids (Fig. 6A). Furthermore, hibernating brown bears had significantly (*p* < 0.001) lower choline concentrations than felids and CKD 3 patients (Fig. 6B). Whereas TMAO was undetectable in hibernating brown bears, felids and CKD 3 patients had significantly (*p* < 0.001) higher TMAO levels (Fig. 6C).

**Table 2:** Betaine, choline, TMAO and renal markers in CKD-susceptible felids and CKD-protected hibernating bears in comparison to CKD stage 3 patients.

N	Captive lions	Captive tigers	Hibernating free-ranging brown bears	Patients with CKD stage 3	*p*
	11	11	15	30	
Age (years)	5.0 (3.0)	2.5 (1.5)	3.2 (1.1)	58.7 (31.1)^a,b,c^	**< 0.001**
Sex (male/female)	6/5	7/4	4/11	22/8	**0.028**
Body weight (kg)	155 (33)	110 (30)	48 (24)^a,b^	78 (20)^a,c^	**< 0.001**
Body temperature (°C)	38.5 (0.9)	40.3 (1.7)	32.9 (2.0)^a,b^	–	**< 0.001**
Creatinine (µmol/l)	175 (81)	173 (41)	206 (50)^b^	173 (55)^c^	**0.008**
Urea (mmol/l)	8.5 (2.9)	7.6 (1.8)	3.5 (5.0)	14.3 (4.0)^c^	**< 0.001**
Betaine (ng/µl)	7.9 (2.5)	7.5 (1.0)	47.7 (36.5)^a,b^	8.6 (4.3)^c^	**< 0.001**
Choline (ng/µl)	9.2 (5.2)	9.2 (33.9)	4.0 (1.4)^a,b^	6.3 (2.6)^c^	**< 0.001**
TMAO (ng/µl)	0.13 (0.16)	0.16 (0.14)	0.00 (0.00)	0.80 (0.90)^a,b,c^	**< 0.001**

Betaine, choline, TMAO, and renal markers in different animal species with a chronic kidney disease (CKD)-like status, e.g. in captive lions, captive tigers, and hibernating, free-ranging brown bears, as well as in patients with CKD stage 3. CKD, Chronic kidney disease; all other abbreviations as indicated in Table 1. Values for median (interquartile range) or total number are shown. Continuous parameters were analysed by Kruskal–Wallis test followed by post-hoc analysis, whereas Chi-squared-test was used for all categorical variables. *p* values for overall group differences are depicted. Letters in superscript indicate *p* < 0.05 as compared to captive lions^a^, captive tigers^b^, or hibernating, free-ranging brown bears^c^ in post-hoc tests.

## Discussion

In the present study, we applied a comparative biomimetic approach to study circulating levels of betaine, choline, and TMAO in healthy humans, CKD patients and different mammalian species, such as captive felids (susceptible to CKD) and free-ranging hibernating bears (protected against CKD). We report substantial differences in betaine, choline, and TMAO levels between species, dietary habits and hibernation status that provide useful insights when designing studies of susceptibility and protection of CKD, and other burden of lifestyle diseases, in relation to human lifestyle and diet (Fig. 2).

The main finding of the study was the exceptional, more than fourfold, increase in circulating betaine observed in free-ranging brown bears during hibernation compared to betaine levels both in the active summer period of free-ranging brown bears, healthy humans and other mammals (Figs. 2 and 3). Generation of betaine during hibernation may have multiple protective effects. During hibernation, bears are typically anuric and it has been reported that their renal blood flow decreases by 36%, while glomerular filtration rate (GFR) declines by 68% compared with the active state^29^. Bears have developed an amazing capacity to protect their kidneys during hibernation, and in the spring renal function returns to normal within a couple of weeks^30,31^. Better understanding of organ protective mechanisms during the vulnerable hibernation period may also provide insights into potential therapies for critically ill patients and organ injury during cold storage and reimplantation during transplantation^32^.

Betaine is an anti-inflammatory and anti-oxidant essential osmoprotective nutrient involved in liver function, cellular reproduction, and cell volume regulation^33^. Since a high-quality genome assembly shows that anti-inflammatory pathways are activated in hibernating bears^34^, the anti-inflammatory potential of betaine in CKD deserves further study. In free-eating healthy human adults there is a negative association between intake of betaine (and choline) and the inflammatory process^35^. As methyl groups are important for numerous cellular functions, such as DNA methylation, phosphatidylcholine synthesis and protein synthesis, the methyl-donor properties of betaine have clinical implications. Moreover, since betaine enhances lipid metabolism and improves insulin resistance in laboratory mice (*Mus musculus f. domesticus*) fed a high-fat diet^36^, high betaine levels may play a role in the healthy obese phenotype^37^ observed in bears preparing for hibernation^38^. As betaine has protective effects on isoprenaline-induced renal failure in rats (*Rattus norvegica f. domesticus*) via a decrease in tumour necrosis factor and nitric oxide synthase^39^, high betaine levels may also protect kidneys during hibernation. Our observation of lower betaine levels in CKD (Fig. 2) and the finding that betaine inhibits cell proliferation and extracellular matrix deposition^40^ supports a renal protective effect for betaine. During hibernation bears should be at high risk (physiologic kidney insufficiency + no intake of B-vitamin) of hyperhomocysteinemia; an established risk factor for CV, cerebrovascular and neurodegenerative disease^41^. However, as we observed no significant increase in homocysteine levels during hibernation (Table 1), high betaine may protect against hyperhomocysteinemia by re-methylation of homocysteine^42^. Accordingly, homocysteine does not elevate in bat (*Myotis pilosus*) brains during torpor due to increased expression of betaine-homocysteine S-methyltransferase^43^. As betaine is an osmolyte that protects proteins, cells and enzymes from environmental stress, such as extreme temperature and lack of water in animals^44^ and plants^45^, betaine may have protective effects when organisms are under environmental stress.

It has been reported that betaine improves animal performance and increases lean body mass, meat quality, and reduces fat deposits in meat-producing cattle, pigs and poultry^18,46,47^. Thus, high betaine during hibernation may protect the bears’ lean body mass and enhance lipolysis, important for energy supply. In humans, high dietary choline and betaine intake have been significantly associated with favourable body composition^48^. The impact of the endogenous microbiota may be significant in this respect in man. While humans have a synthetic capacity for betaine, this is supplemented via nutritional acquisition of substrates and subsequent conversion to betaine by gut microbes. Seasonal changes in the gut microbiota in bears, with the ability to transfer seasonal metabolic characteristics to germ-free mice via fecal transplants have previously been reported^49^. As choline and other methyl donors regulate methylation of genes related to memory and cognitive functions^50^, high betaine and choline levels may also protect their brain during hibernation. In addition to serving as an osmolyte and metabolic intermediate, betaine influences pathways of inhibitory neurotransmitter production-recycling^51^.

The reason(s) why free-ranging bears can give birth and lactate (conditions associated with high metabolic demand) during hibernation^52^, are likely physiological and behavioural adaptations through which offspring survive harsh seasonal conditions, such as inclement weather or low food availability^53–55^. The successful development of the fetus depends on optimal dietary intake of folate, choline and betaine involved in one-carbon transfer methylation and related epigenetic effects on gene expression^56^. Moreover, maternal and postnatal dietary methyl nutrients have effects on the epigenetic machinery that in adulthood may lead to non-communicable diseases^57^. As the supply of methyl groups is vital during pregnancy, we speculate that high levels of betaine protect the fetuses in pregnant bears during hibernation. Indeed, betaine plays an important role in physiologic pregnancy^58^, fetal development^59,60^ and DNA methylation^61^. Furthermore, in dairy cattle, betaine supplementation increase lactation performance^62^.

The mechanism(s) by which betaine and choline levels increase during the long period of fasting are unclear. However, in part, the increase may reflect an excessive intake of betaine-rich food in the autumn in preparation for hibernation. As betaine is found in microorganisms, plants, and animal muscle, it is a significant component of many foods. The daily intake of betaine in the human diet ranges from an average of 1 g/d to a high of 2.5 g/d. Nutrients rich in betaine include wheat bran (1339 mg/100 g), wheat germ (1241 mg/100 g), spinach (645 mg/100 g), shrimp (218 mg/100 g), and wheat bread (201 mg/100 g)^63^. Although some bears in our study area do eat wheat during spring and early summer^64^, betaine-rich foods are not part of the typical diet of free-ranging Scandinavian brown bears, which on an annual basis usually consists of 50% protein (ants, ungulate carcasses) that is mainly consumed in spring, and 50% carbohydrates (berries) that is mainly consumed in summer and fall^65^. The betaine content in bilberries is rather low (0.2 mg/100 g)^63^. However, since free-ranging bears during hyperphagia can eat a massive amount of different berries per day a significant amount of betaine will be ingested in the peak season. As increased intake of betaine- and choline-rich food in the autumn is unlikely to increase circulating betaine as much as fourfold, a metabolic switch that increase endogenous production of choline is likely to occur before and under hibernation. Since ≈30% of phosphatidylcholine can be produced by the sequential methylation of phosphatidyl-ethanolamine (via PE-methyltransferase), the body has the capacity to make choline ´de novo’^66^. Similarly, Olthof et al.^67^ have shown that supplementation of choline, as phosphatidylcholine, increases the pool of choline for the production of betaine. Thus, since phospholipids and phosphatidyl-ethanolamine could be used as an endogenous source for choline, further studies need to investigate these metabolic events in hibernating mammals^68^.

Beside bears, only few mammals and birds are known to give birth and lactate during hibernation^69^. To investigate if high levels of betaine during hibernation are specific for bears or a feature of temperature changes, we studied garden dormice—another hibernating species. In contrast to bears’ constant hibernation body temperature, dormice have to periodically rewarm from cold torpid states to warm interbout intervals^70^. We find that whereas betaine and choline are not generated in dormice at 5 °C they are rapidly generated at 37 °C despite fasting (Fig. 4). This supports the hypothesis of a metabolic switch with endogenous betaine production as cell-protective substance during warming-up periods. The torpor-arousal switch is characterized by the accumulation of metabolites of nitrogen (glutamine) and phospholipid (betaine) catabolism in late torpor with the capacity to act as protective osmolytes^71^.

In contrast to free-ranging hibernating bears, which are protected against CKD and the CKD complications observed in the human uremic phenotype, such as osteoporosis, sarcopenia and arteriosclerosis^30^, wild and domestic felids are susceptible to CKD^72^ and arteriosclerosis^73^. The prevalence of CKD in domestic cats (*Felis catus*) is high (estimated to affect 1/3 of older cats) and has increased in recent decades^72^. The tubulointerstitial changes, including fibrosis, that are present in the early stages of feline CKD become more severe in advanced disease^72^. As transforming growth factor (TGF)-β1 is involved in epithelial cell de-differentiation, growth arrest and apoptosis in feline CKD, it has been suggested that feline CKD is a useful, naturally occurring model of human CKD^74^. The prevalence of CKD in wild felids is not well studied. However, a recent thesis concluded that CKD is a prevalent disease affecting non-domestic felids throughout zoos in Australia^75^. In accordance, a study of captive wild felids at German zoos, showed that renal lesions were detected in 33 out of 38 animals^76^. A variety of factors—including ageing, ischemia, comorbidities and high phosphorus overload due to a carnivorous diet have been implicated as factors that promote felid CKD^72^.

Gut microbial metabolism of dietary choline, a nutrient abundant in the Western diet, produces TMA and the atherothrombosis- and fibrosis-promoting metabolite TMAO. High TMAO levels are mechanistically linked to atherosclerosis^77^ and increase the risk for premature CVD^78^. The high TMAO levels reported in CKD are due to both impaired renal function^20^ and gut dysbiosis with higher percentages of opportunistic pathogens^79^. As TMAO promotes interstitial nephritis^80^, we measured TMAO in felids (Fig. 6). High intake of red meat (especially processed and red meat) has been associated with increased risk of CKD^81^ and premature ageing in the general population^82^. Chronic dietary exposures of red meat increase TMAO levels via modulation of the gut microbiota^83^. We compared CKD 3 patients with felids and hibernating brown bears with comparable levels of S-creatinine (Table 2) and report major differences in betaine and TMAO levels (Fig. 6). Both fasting^84^ and hibernation^85^ alter the gut microbiota. As TMAO was not detectable in free-ranging active bears (except one) this suggests that free-ranging brown bears do not synthesize TMA due a “healthy” gut microbiota. It is noticeable that of the eight species of bacteria from two main phyla (*Firmicutes* and *Proteobacteria*) that use choline for production of TMA^86^ none was reported in a study of bear microbiota composition in relation to seasonal changes in energy metabolism^49^. TMAO promotes vascular calcification^87^ and the absence of TMAO in free-ranging bears may contribute to their low risk of arteriosclerosis despite massive hypercholesterolemia^68^.

In contrast to free-ranging bears, TMAO was measurable in all captive bears, likely related to different food habits associated with altered gut microbiota. As captivity and domestication have dramatically reshaped the gastrointestinal microbiota in numerous species, e.g. in wild and domestic equids^88^, free-ranging and captive Andean bears (*Tremarctos ornatus*)^89^, and primates^90^, loss of diversity (and maybe infection of human microbiota from animal keepers) most likely occurs in captive brown bears, which contributes to the generation of TMAO. Higher *Firmicutes* to *Bacteroidetes* enrichment leads to a greater response to dietary TMAO precursor intake^91^. Thus, differences in microbial composition between humans and the other species likely contribute to the observed difference. As fermented food lowers TMAO levels in healthy adults^92^, a difference in the amount of ingested fermented food could also contribute to observed differences. To the best of our knowledge, circulating TMAO levels have not previously been measured in a comparative human–animal study. However, high levels of TMAO in muscle tissue have been reported in marine mammals^93^. As muscle TMAO contents increase linearly with depth in six fish families^94^, it has been suggested that TMAO counteracts protein-destabilizing forces in deep-sea water. We report that circulating TMAO levels are higher in omnivorous healthy humans compared to other species (Fig. 2C). As humans are the only species in this study that eats fish on a regular basis, this could contribute to higher TMAO levels. Plasma TMAO concentrations were directly associated with dairy consumption and low-grade inflammation^95^ and in vegans/vegetarians the transformation into TMA via low-abundance microbiota occurs at a markedly lower extent compared to omnivores^96^.

The results of this descriptive study should be interpreted taking a number of caveats into account. Firstly, the number of healthy controls, CKD patients and animal species are limited. However, even with this rather limited number we have observed major differences between the species. Our sample numbers were, however, too small to enable sex-specific statistics. Secondly, the free-ranging and captive bears were not matched for weight and age. Thirdly, as we do not have information on dietary habits in healthy controls, CKD patients and free ranging bears we cannot relate our findings to dietary exposures. Our study would also have benefited by faecal or circulatory microbiota cultures in the different species. Creatinine, a by-product of muscle catabolism excreted by the kidneys, is considered a reliable index for kidney function. However, as S-creatinine is also affected by muscle mass and meat intake it is possible that higher muscle mass and the carnivorous diet contributes to high S-creatinine levels in tigers and lions.

In conclusion, this comparative biomimetic study suggests that a metabolic switch leading to endogenously produced betaine protects bears and garden dormice during the vulnerable hibernation period. Our data also show that carnivorous eating habits in the animal kingdom are linked to higher TMAO levels. Furthermore, we show a strong association between body temperature and the endogenous production of betaine and the depletion of TMAO. The characterization and manipulation of a metabolic switch that divert choline to generation of betaine instead of TMAO would likely have a major impact in prevention of not only CKD but also other burden of lifestyle diseases.

## Materials and methods

### Chronic kidney disease patients and healthy controls

Fasting plasma samples from Caucasian CKD stage 3 (eGFR 30–60 ml/min; n = 30) and stage 5 (eGFR < 15 ml/min; n = 121) patients, consecutively recruited into two observational prospective cohort studies, were analysed for betaine, choline, and TMAO. Part of these data have previously been published^20^. Exclusion criteria were age < 18 years, active hepatitis B/C, HIV or signs of acute infection. The Ethics Committee of the Karolinska Institutet approved the study protocol (244/01, 2007/1663-31/4, 2016/1470-31/4) and informed written consent was obtained from all patients. Eighty age- and sex-matched population-based individuals in the Stockholm region of Sweden, randomly selected by Statistics Bureau of Sweden (www.scb.se) served as controls. No other exclusion criteria than unwillingness to participate in the study were applied in the selection of the healthy controls.

### Animal cohorts:

#### Free-ranging brown bears (*Ursus arctos*)

The Scandinavian Brown Bear Research Project is a long-term individual-based ecological study of brown bears in central Sweden. All animal handling and sampling was approved by the Swedish Ethical Committee on Animal Experiments (application numbers C212/9 and C47/9) and the Swedish Environmental Protection Agency. Study protocols and procedures have been described previously^31^. Briefly, blood samples were obtained from 15 free-ranging, sub-adult, 2- to 3-year-old Eurasian brown bears (*Ursus arctos*) (11 females, mean body weight in winter: 47 kg; four males, mean body weight in winter: 35 kg) in Dalarna County, Sweden, 2010–2012. For this purpose, each bear was captured during hibernation, i.e. in February or March, as well as during the active period in June of the same year. Bears were anesthetized by darting with a combination of tiletamine–zolazepam (1.1 mg/kg), medetomidine (0.03 mg/kg) and ketamine (hibernating animals) or tiletamine–zolazepam (4.7 mg/kg) and medetomidine (0.09 mg/kg) (active animals)^31,97^. All blood samples were taken from the jugular vein and approximately 1 h after sampling, blood was centrifuged and subsequently immediately frozen on dry ice until storage at − 70 °C^31^.

#### Captive brown bears

The captive European brown bears were sampled during regular veterinary health checks. The animals were housed in large, naturalistic enclosures (> 5.000 m^2^) in Germany, Poland, Kosovo, and Sweden. Only blood samples from healthy individuals were included in this study. Their diet consisted, besides foraging of grass in the enclosure, mainly vegetables, fruits, eggs, and nuts. Occasionally the animals received wild ungulate meat, dog-pellets, berries, and bread. Sampling took place under general anesthesia with ketamine (2.2 mg/kg), medetomidine (0.035 mg/kg), midazolam (0.05 mg/kg), and butorphanol (0.05 mg/kg), after being starved for 10–12 h, with water ad libitum until injection. The jugular vein was used for fasting blood sampling in all 34 bears (18 females, mean body weight: 150 kg; 16 males, mean body weight: 234 kg, age-range 2–16 years old).

#### Garden dormice

The animals involved were part of a different study on hibernation^98^ (Austrian Federal Ministry of Education, Science and Research license number 68.205/0137-WF/V/3b/2014) and reviewed and approved by the institutional ethics committee of the Veterinary University in Vienna, Austria and the relevant Austrian authority in accordance with the Austrian Animal Experimentation Act. The garden dormice issued from a breeding colony were kept at the Research Institute of Wildlife Ecology (Vienna, Austria). They were housed in cages equipped with one nest, bedding and nesting material, and kept under natural fluctuations of ambient temperature and photoperiod. During hibernation, they were kept at 4 °C in ventilated cooling units within a customized nest and bedding material under constant darkness. During pre-hibernation period the animals were fed protein pellets (commercially available, processed cat food pellets) enriched with poly-unsaturated fatty acids (PUFAs), linseed, and safflower oil. Fasting blood sampling took place post-mortem from the heart. About 23 dormice (mean body weight: 118.2 g) were sacrificed in active status, whereas 23 different animals (mean body weight: 107.0 g) were investigated in torpor status of hibernation (N = 14 early torpor; N = 9 late torpor), as well as 19 other dormice (mean body weight: 105.4 g) that were euthanized in interbout interval during hibernation (N = 11 early interbout interval; N = 8 intermediate interbout interval).

#### Captive lions and tigers

Twenty-two animals (11 lions and 11 tigers) were sampled during standard, random veterinary health checks and only healthy individuals were included in the study. The individuals were housed in zoological parks in Europe and fed a standard large carnivore diet consisting of mainly red meat (beef and horse meat), bones, occasional white meat (chicken), and calcium powder. Six male (mean body weight: 178 kg) and five females (mean body weight: 148 kg) lions, as well as seven male (mean body weight: 110.6 kg) and four female tigers (mean body weight: 124.3 kg), were available for the present analysis. Mean age of the lions and tigers was 4.9 and 3.2 years, respectively. All animals were immobilized with a combination of 3.5 mg/kg ketamine, 0.035 mg/kg medetomidine, 0.1 mg/kg midazolam, and 0.05 mg/kg butorphanol. Fasting blood sampling was obtained from the jugular vein. None of the lions and tigers were pregnant at the time of blood sampling and no contraceptive methods were used. All use of samples from tigers and lions were reviewed and approved by the institutional ethics committee of the Veterinary University of Vienna, Austria.

#### Wild boars (*Sus scrofa*)

The animal study was reviewed and approved by the institutional ethics committee of the Veterinary University in Vienna, Austria and the relevant Austrian authority in accordance with the Austrian Animal Experimentation Act (Austrian Federal Ministry of Education, Science and Research license numbers 68.205/0224-WF/V/3b/2016 and 68.205/0159-WF/V/3b/2016). Animals were kept under naturalistic conditions in a large acorn forest and supplementary fed with ~ 1.2 kg/animal/day of corn. Details on housing and handling of the animals have been described previously^99^. Ten 6-year-old, non-pregnant females were available for the present study. The animals were immobilized using a combination of 3.2 mg/kg tiletamine–zolazepam, 0.07 mg/kg medetomidine, and 0.15 mg/kg butorphanol.

### Assays

All methods were performed in accordance with relevant guidelines and regulations for humans and animals. In human subjects, plasma heparin samples were obtained after a 12 h fast and stored at − 80 °C prior to measurements. In brown bears, garden dormice, captive lions and tigers, as well as wild boars, blood samples were taken as indicated in the respective section. Quantification of betaine, choline, and TMAO was performed by Liquid chromatography with tandem mass spectrometry with a previously published protocol in a 96-well format^22^. In more detail, plasma aliquots were spiked with internal standards, comprised of TMAO-D9, betaine-D9, and choline-D9 in 80% methanol. The metabolite extract was collected, 20 µL was transferred to a 96 well plate, and evaporated to dryness. Before analysis, the samples were dissolved in 100 µL 50% MeOH with 13C5-proline as a recovery standard, and subsequently injected into an Agilent 1290 Infinity chromatographic system (Agilent Technologies, Waldbronn, Germany) fitted with an Acquity UPLC Amide column in combination with a VanGuard precolumn (Waters Corporation, Milford, MA). Detection of the metabolites was performed using an Agilent 6490 Triple Quadrupole mass spectrometer (Agilent Technologies, Santa Clara, CA). Data processing was done by MassHunter Quantitative Analysis QQQ (Agilent Technologies Inc. Santa Clara, CA). Importantly, we have previously demonstrated that metabolite levels remain stable and are comparable when using either plasma heparin and serum samples and multiple number of freeze–thaw cycles^22^. All other parameters in the free-ranging bears, as well as in the captive tigers and lions, were quantified in our in-house lab (Karolinska Institutet, Stockholm, Sweden). Insulin was measured using an enzyme linked immunosorbent assay according to the manufacturer’s instructions (Iso-Insulin, #10-1128-01, Mercodia, Uppsala, Sweden). Cystein, cysteinylglycin, and homocystein levels were measured by HPLC as modified from Ubbink et al.^100^. Routine parameters, including albumin, total protein, total cholesterol, triglycerides, liver enzymes, markers of renal function, and electrolytes were measured using a fully automated, random access analyser (Konelab, Thermo Fisher Scientific, Vantaa, Finland).

### Statistical analysis

SPSS software version 26.0 (IBM, Armonk, NY) and GraphPad Prism 8 (GraphPad Software, San Diego, CA) were used for all statistical analyses. Overall group differences between the three human subgroups (healthy subjects, CKD 3 subjects, CKD 5 subjects), between different species and diets, as well as between animals with CKD-like status (hibernating bears and felids) and human patients with CKD, were assessed by non-parametric Kruskal–Wallis test with Bonferroni post hoc analyses for group-wise comparisons, respectively. Longitudinal data in the Scandinavian Brown Bear Research Project on hibernation compared to non-hibernation periods were analysed using non-parametric Wilcoxon signed-rank test. Comparisons between captive and free-ranging bears were calculated using non-parametric Mann–Whitney U test. A *p* value of < 0.05 was considered as statistically significant in all analyses.

## Data Availability

The datasets generated during and/or analysed during the current study are available from the corresponding author on reasonable request.
